# The Development of Chinese Assessment and Evaluation of Physical Literacy (CAEPL): A Study Using Delphi Method

**DOI:** 10.3390/ijerph17082720

**Published:** 2020-04-16

**Authors:** Si-Tong Chen, Yan Tang, Pei-Jie Chen, Yang Liu

**Affiliations:** 1 School of Physical Education and Sport Training, Shanghai University of Sport, Shanghai 200438, China; sitongchen@szu.edu.cn (S.-T.C.); tangyan@sus.edu.cn (Y.T.); 2 Shanghai Research Center for Physical Fitness and Health of Children and Adolescents, Shanghai University of Sport, Shanghai 200438, China; chenpeijie@sus.edu.cn; 3 School of Kinesiology, Shanghai University of Sport, Shanghai 200438, China

**Keywords:** assessments and evaluation, CAEPL, children and adolescents, China, physical education, physical literacy, theoretical model

## Abstract

The aim of this study is to introduce the development of the Chinese Assessment and Evaluation of Physical Literacy (CAEPL), to present the theoretical model of the CAEPL, and to quantify the weight of each domain of the CAEPL. 34 experts took part in the Delphi survey, and 23 experts completed all the three rounds of the survey. Experts’ opinions are used to develop the theoretical model of the CAEPL. The analytical hierarchy process (AHP) was employed for determining the weights of subdomains and items of the CAEPL. The CAEPL is a comprehensive model, including intention of physical activity (IPA), knowledge of physical activity (KPA), motor/sport skill (MSS), behavior of physical activity (BPA) and physical fitness (PF). Specific weights of IPA, KPA, MSS, BPA and PF are 17.25%, 16.23%, 27.01%, 23.72% and 15.79%, respectively. The CAEPL provides an important and useful instrument to measure and improve physical literacy (PL) among young Chinese people. Studies on the feasibility, reliability, validity and sensitivity of the CAEPL should be conducted to improve it in the future.

## 1. Background

In the 1980s, children and adolescents in the UK were struggling in physical inactivity (PIA) and misguided physical education (PE). Furthermore, the flourishment in existentialism and phenomenology emphasized the importance of embodiment. Accordingly, a British PE researcher, Margaret Whitehead, proposed the concept of physical literacy (PL) [1]. She defined PL as ‘motivation, confidence, physical competence, knowledge and understanding that individuals develop in order to maintain physical activity at an appropriate level throughout their life’ [1]. Furthermore, this definition was modified into ‘the motivation, confidence, physical competence, knowledge and understanding to value and take responsibility for engagement in physical activities for life’ issued by the International Physical Literacy Association (IPLA) in 2013 [2]. Hereafter, the concept of PL has gained worldwide attention [3,4], largely due to its potential benefits in innovating PE [5,6,7], promoting being active [8,9] and optimising health [8,10,11]. Some countries have devoted themselves to enhancing PL in children and adolescents. For example, national programs incorporating the concept of PL have been launched in Canada, the UK and the US [12].

Assessments and evaluations of PL have attracted research interest [3,13]. So far, three assessments of PL have been established and are used widely: the Canadian Assessment of Physical Literacy (CAPL) [13,14]; Passport for Life (P4L) [15]; and the Physical Literacy Assessment for Adolescents (PLAY) [16]. These three instruments to assess PL exhibited similarities and differences. In general, these assessments targeted children and adolescents as testing subjects, using both objective and subjective measures to assess participants’ fitness, knowledge, behaviors, competence and affection [13,14,15,16]. However, owing to difference aims to assess PL, these three assessments have their own features of evaluating participants’ performance of PL, selecting assessors, test time duration and facilities. Although those assessments unlocked new insight into understanding PL [17], the assessments were debated for some of their limitations [18], such as fidelity to concept and the testing burden. Besides, a few other western countries such as Australia and the US are attempting to develop PL assessments and evaluations for children and adolescents [19,20,21].

Over the past three decades, Chinese children and adolescents underwent significant declines in physical fitness (PF) and health [22], partially owing to insufficient physical activity (PA) and sedentary lifestyles [23,24]. Consequently, Chinese policymakers have designed national plans and strategies to cope with the health crisis [25,26], one of which was to establish systematic health-related behavior monitoring and surveillance [27,28]. In September 2019, the State Council the People’s Republic of China (SCPRC) issued a circular to develop China into a leading sports power. This national strategic plan points out that an increase in PL is one of the strategic goals of health promotion in China [29]. In addition to health promotion, educational sectors in the country have been implementing comprehensive education reforms, including incorporating PL as a main goal of school PE [30], addressing perceived shortcomings in ‘Gaokao’ (National College Entrance Exam, NCEM) and providing an emphasis on a more well-rounded education system and the development of individual competencies [31]. In the face of such demands and goals, it is believable that adopting PL can be an innovative approach attempting to solve the crisis of PIA and health, as well as educational forms [32,33]. It is therefore necessary and urgent to develop an assessment of PL among Chinese children and adolescents.

Over the past five years, as one of the leading institutes in sport and health science in China, Shanghai University Sport (SUS), has initiated a study on how to assess PL among Chinese children and adolescents, and developed the Chinese Assessment and Evaluation of Physical Literacy (CAEPL). Hence, the aim of this study is to present the development of the CAEPL theoretical model for school-aged children and adolescents in China, and identify the weights of CAEPL’s domains and their indicators for assessment.

## 2. Methods

To develop the Chinese Assessment and Evaluation of Physical Literacy (CAEPL), a literature review sought expert consultations and utilized the Delphi Method, and the analytical hierarchy process (AHP) method was also undertaken. The study protocol was approved by Ethics Review Committee of Shanghai University of Sport (#2017037)

### 2.1. Contextualizing and Operalizaing the Concept of PL in Chinese Context

Chinese researchers have interpreted the concept of physical literacy (PL) by Whitehead [28,34] in the context of China. The most widely used interpretation derived from Chen et al. [28], who proposed an adaptive definition of PL as a comprehensive capability integrating different components that benefit individual active lifestyles and health throughout lifespan. Based on previous published assessments of PL [13,14], the theoretical assessments and evaluations of the CAEPL have five domains: (1) intentions of physical activity (IPA); (2) knowledge of physical activity (KPA); (3) behaviors of physical activity (BPA); (4) motor/sport skills for physical activity (MSS); and (5) physical fitness (PF, which can be understood as an outcome of PA). This theoretical model can be corroborated by preceding theories on assessments of PL, as key domains of PL are involved in affective, cognitive, behavioural and fitness dimensions, which is in line with the current model.

### 2.2. Delphi Process to Develop the CAEPL

**Figure 1:**
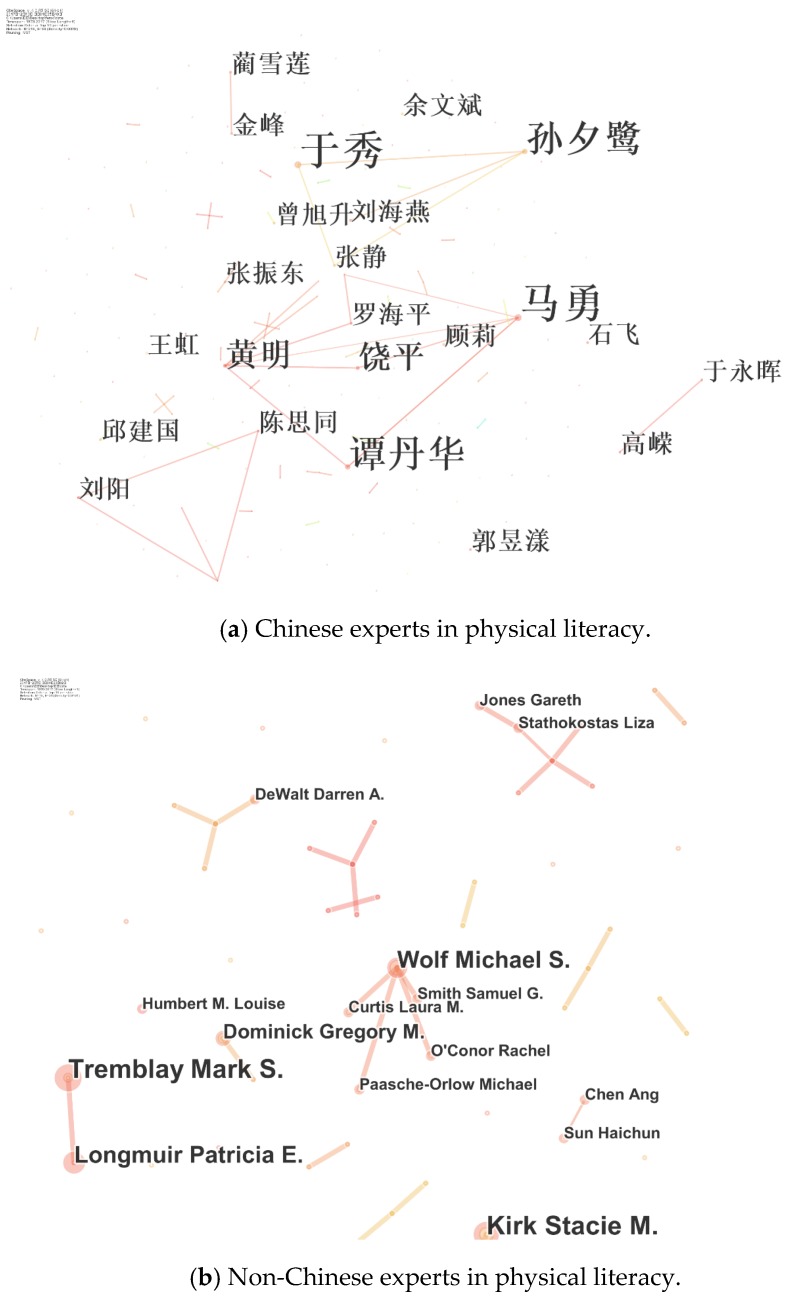
The selected experts participating in the Delphi process by CiteSpace.

Using a Delphi process, expert opinions on the CAEPL model and the relative importance of each item were collated. This anonymous process used three rounds of controlled feedback, limiting the influence of comments from peers. This study was approved by the Research Ethics Board of Shanghai University Sport (SUS). International and Chinese experts were identified by an electronic academic database. Recommendations expanded the list of potential experts to ensure that representation for each CAEPL domain was considered by the original expert panel. All identified experts were invited to participate in the three-round Delphi process. To identify experts accurately, we used CiteSpace (v. 4.0.R5 SE, https://sourceforge.net/projects/citespace/) to help search experts in the field of PL (Figure 1). However, some experts filtered by CiteSpace cannot meet our criteria, since their studies were not high-quality. Hence, based on screened results, we selected three Chinese experts (Yu, Liu and Chen S.T.) and four international experts (Tremblay, Longmuir, Sun and Chen A). In addition to this, manual filters to identify experts were conducted through the authors’ consultations and discussions.

#### 2.2.1. Procedures of the Delphi Process

**Figure 2:**
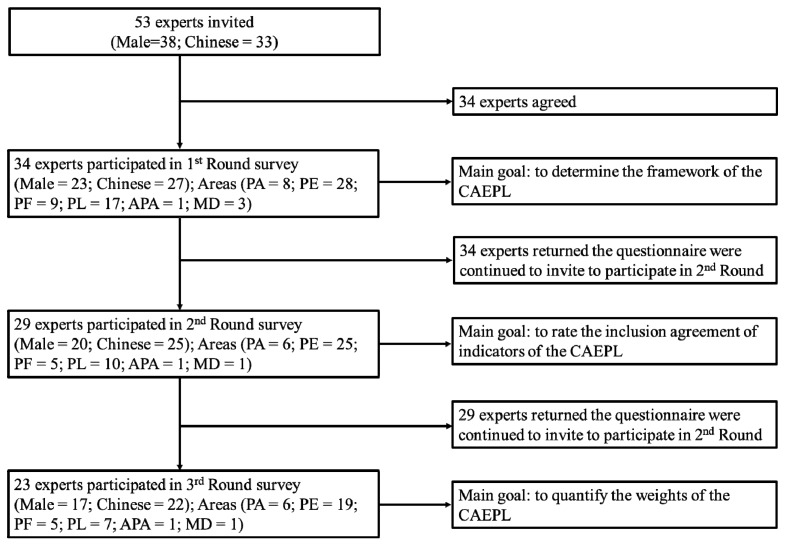
The procedure of Delphi process. PA—physical activity; PE—physical education; PF—physical fitness; PL—physical literacy; APA—adapted physical activity; MD—motor development; CAEPL—Chinese Assessment and Evaluation of Physical Literacy

The goal of the Delphi process is to reach a consensus on a specific research issue after multiplied rounds of discussions. For this present study, a consensus was defined as agreement among 75% or more of the panel [35]. Each round was implemented by a webpage and reminder e-mails from November 2017 to March 2018. Experts not responding were withdrawn from the subsequent rounds. The Delphi procedures can be seen in Figure 2. In brief, the first round was used to examine the theoretical model of the CAEPL. The goal was to collect experts’ opinions regarding the proposed domains of the theoretical model. Using the Five Likert-scale (very agree,…, very disagree), each expert rated whether the proposed domain should be included in the theoretical model. Experts’ opinions and feedback were analysed by three independent researchers (S.-T.C., Y.L. and Y.T.) For the second round, the remaining Delphi participants were asked to rate each sub-domain and its statements using the Five Likert-scale. Sub-domains and statements with 75% agreement by experts were included in the model. For some statements with agreement of less than 75%, three independent researchers (S.-T.C., Y.L. and Y.T.) judged the feedback from experts and determined whether the statements were excluded or included. Lastly, for the third round, experts were invited to determine the relative importance of domains of PL, sub-domains and their statements. The AHP was employed for determining the weights of subdomains and items of the CAEPL.

#### 2.2.2. Other Complementary Developments

In brief, the KPA questionnaire was developed by SUS and Shanghai Municipal Education Commission (SHMEC) through multi-stage discussions. According to China’s current PE curricula standard, the experts panel determined five KPA questionnaires for school-aged children. Furthermore, researchers developed the IPA scale. To develop the MSS assessment, researchers have developed a specific sport events skills assessment involved in 12 specific sports events (e.g., basketball, soccer and volleyball). Regarding the domain of PF, our study applied the National Student Physical Fitness and Health Test as it is a nationally recognized test in China, albeit with some limitations.

#### 2.2.3. Using AHP to Determine Weights of the CAEPL

The AHP was adopted to determine the specific weights of the CAEPL. First, the CAEPL was stratified and set up a goal tree, where the indicators within each dimension were stratified to form a hierarchical structure and a weight evaluation goal tree was constructed. Next, we set up judgment matrices. According to the hierarchical structure of the goal tree, we built up pair-wise comparisons of the judgment matrices to define the relative importance of each individual indicator, and designed a weight questionnaire for expert rate. For the expert panel, experts of the third round of the Delphi process were recruited. The expert panel enumerated the judgment matrices in the questionnaire according to the CAEPL weights, and marked the goal tree based on pair-wise comparisons. For indicators at the same level, a certain weight was individually given according to its value on the indices at the upper level. Yet Another AHP (YAAHP) software (Yuanjuece Software and Technology Ltd., Taiyuan, China) was applied to the expert scoring process. The consistency of the expert judgment matrix was calculated to test the logic of the judgment. Generally, when the consistency index (CI) was less than 0.10, the judgment matrix was strongly consistent, and the weight coefficient met logic consistency.

## 3. Results

Table 1 showed the characteristics of experts across each round in the Delphi process. Of 53 experts invited to participate in this study, the majority were male and from China; 45 experts were physical education practitioners. 34 experts agreed to participate in the first round and returned their responses, while seven came from North America and Europe. Subsequently, 29 experts returned their responses after completing the second round, which included only four non-Chinese experts. Of the third round, 22 Chinese experts involved in physical activity (PA), physical education (PE), physical fitness (PF), physical literacy (PL), adapted PA and motor development completed the analytical hierarchy process (AHP) evaluation. As a result, 53 experts from across the world were invited to participate in this study, of which 23 completed three rounds (*n* = 23, response rate = 43.4%).

Due to the current limitations, we cannot report all the detailed information of the Chinese Assessment and Evaluation of Physical Literacy (CAEPL), but the framework of the CAEPL theoretical model, as well as the weights of its five main domains. Table 2 shows the theoretical model for the CAEPL and the agreement (%) of the five main domains. In general, all the domains, sub-domains and the specific aspects reached agreement of more than 75% except ‘active transportation’, ‘screen-based time’ and ‘homework time’. However, after inductive analysis, these three specific items should be included in the model for their rationale in promoting health in children and adolescents. The results of AHP show that the weights of intention of physical activity (IPA), knowledge of physical activity (KPA), behavior of physical activity (BPA), motor/sport skill (MSS) and PF were 0.1725, 0.1623, 0.2372, 0.2701 and 0.1579, respectively (Table 3). Among the five main domains, different sub-domains with weights are presented. Owing to some limitations of study protocol of the CAEPL, this study cannot report the weight of indicators of the CAEPL. Those indicators comprised internationally recognized and Chinese-adaptive measures. For example, when assessing MSS, two components, including fundamental movement skills (FMS, internationally recognized) and specific sports-event skills (Chinese-adaptive), were included. The consistency index (CI) was 0.0033 (less than 0.10), meaning the judgment matrix was strongly consistent, and the weight coefficient was logically consistent and acceptable.

## 4. Discussion

To our knowledge, the present study is to first develop the theoretical Chinese Assessment and Evaluation of Physical Literacy (CAEPL) model and the CAEPL protocols within each domain, as well as individual indicators among Chinese children and adolescents. In this study, we primarily established the theoretical CAEPL model and determined its specific weights. This study plays an important role in advancing the assessment and evaluation of physical literacy (PL), which can share China’s experience with researchers across the world.

Having some supportive conditions to develop the CAEPL, we expected that the CAEPL might be generalized into China for improvements in assessing the effects of school physical education (PE), which fills the gap in the previous research and practices of there being no appropriate or comprehensive assessment for combinations of behavior, knowledge, fitness, affective and psychology for Chinese children and adolescents. To the authors’ knowledge, the CAEPL is relatively one of the most advanced comprehensive assessment and evaluations of PL for children and adolescents in China. Its advent may benefit Chinese educational reforms, promote active lifestyle, implement PL education and improve school PE in the country. In China, health promotion and the promotion of healthy lifestyles by fostering PL is a novel research issue. As discussed by Dudley and his colleagues [19], fostering PL should align with relevant policies at the national and regional levels. Dudley and colleagues [19] proposed four main pillars to foster PL in the fields of public health, recreation, sport and education, namely competencies, contexts, journeys and power structures, which established a policy-oriented framework to develop PL in western countries. However, owing to largely varied social and physical environments, social norms and local cultures across diverse nations or regions, the framework proposed by Dudley et al. [19] may not be suitable. In order to address the practical issue of how to foster PL in different countries, policy environment in the country is a necessary consideration.

To our knowledge, this is one of very few assessments of PL for children and adolescents in China. Compared with Yu et al. [36], the present study applied more standardized protocol to develop the assessment and evaluation of PL, making the instrument more reliable. Moreover, we added an overall indicator rating the PL level, filling the gaps in previous Chinese studies [27]. Furthermore, the CAEPL was developed based on international experts, which paves the foundation for wider international generalizations and applications. It is the first assessment and evaluation of PL for children and adolescents that was reported internationally in China. In this study, our concept of PL was in part rooted from the Whiteheadian concept that was used predominantly across the world. Even as we contextualized the Whiteheadian concept into a Chinese context cross-culturally, the CAEPL, based on the Chinese concept of PL, still has the possibility to be generalized globally. Thus, when compared with other assessments like the Canadian Assessment of Physical Literacy (CAPL), Passport for Life (P4L) and Physical Literacy Assessment for Adolescents (PLAY), caution is required.

When assessing PL among children and adolescents, the CAEPL applies a questionnaire to assess participants’ intention of physical activity (IPA), which was based on international well-established measures. For the IPA questionnaire, 20 items on a five Likert-Scale were developed for assessment and evaluation. With regard to knowledge of physical activity (KPA), the knowledge questionnaire involved in five aspects was developed, which has multiple questions (grade 1–2: 30; grade 3–4: 50; grade 5–6: 60; grade 7–9: 80; and grade 10–12: 100). In the domain of behavior of physical activity (BPA), objective measures (e.g., accelerometer or pedometer) and subjective measures (e.g., International Physical Activity Questionnaire) are both used to capture various components of physical activity (PA) behavior. Fundamental motor skills assessments, such as the Test for Gross Motor Development-3 (TGMD-3), are an imperative to assess motor/sport skill (MSS). Besides, sport event specific assessments were also developed to assess a participant’s performance in one sport event. To assess physical fitness (PF) in line with national standards, the CAEPL uses the National Student Physical Health Standard. As developers of the CAEPL, we should and must admit that the current form of the CAPL is a theoretical model, implying more improvements should be implemented during the application process. Fortunately, this work has been conducted, and will have been completed in 2020.

As is known to all, the three above-mentioned Canadian assessments have their own traits. One of these is that they could be used for children with a limited age range. However, the CAEPL is tentatively suitable for children with a wider age range (6–18 years), which may be applied more widely than was in the three assessments. The CAEPL is the first one that adds a specific sports-event skills assessment, which other assessments fail to do. It seems somewhat advanced in PL assessment and evaluation. The inclusion of a specific sports-event assessment of the CAEPL is a China-adaptive characteristic, since grasping one or two sports-event skills has been a goal of school PE in China. Thus, this inclusion should be judged whether it is appropriate by robust evidence.

Although the theoretical model of the CAEPL has been established, some considerations derived from the development should be mentioned, which might be beneficial to future studies. Opinions regarding clarifications of the Chinese concept of PL need to be emphasized. As some experts indicated that we should clarify the Chinese concept of PL, the definition of the Chinese concept of PL seems to be argued. Due to the different backgrounds of experts, this argument is conceivable. Future studies should further clarify the Chinese concept of PL. Some experts suggested that sports morals (which can be understood as non-violence, for example, conforming to rules or tactics) should be included [36]. However, how to define the moral of sports or PA setting is still a research issue, indicating that sports morals cannot be directly measured. Hence, it is not recommended to include sports morals. The measurement of PF for Chinese school-aged children is doubted, as some measures have lower validity. For example, in the Students Physical Fitness and Health Test (SPFHT), measurements of 800- and 1000-m runs were used as measures of girls’ and boys’ aerobic capacity, respectively. However, such measures are not well-recognized. Therefore, we should replace these measures with more acceptable measures, such as a 20 m shuttle run [13].

The weights of domains of the CAEPL are different from previous PL assessments developed by Western researchers. For example, the CAPL developed by Canadian researchers [13] had two domains with equal weights (physical competency and daily behavior). The difference can be interpreted by different operationalized ways. In the CAPL, the research operationalized PL into four domains, while five domains were done in the current study. Similarly, however, the CAPL and CAEPL both stressed the relative importance of behavior and skills rather than other domains. It is plausible that the main drivers of better performance in PL in school-aged children are competency and behavior. In addition, backgrounds of Delphi experts should be considered when interpreting our results. Experts were primarily from China in this study; their expertise may influence the relative importance across the domains of the CAEPL. For example, the largest weight across the domains of PL was motor skills, potentially as major experts were from school PE fields. In China, one primary focus of school PE is to develop school-aged children’s and adolescents’ MSS. Hence, it is plausible that MSS shared the largest weight within the CAEPL.

On the other hand, during the Delphi survey, some experts’ opinions should have been taken into consideration. For example, one of Delphi participants suggested that knowledge related to humanity should be included—we admit the importance of this suggestion. However, standing on the perspective of health promotion, humanity-oriented knowledge may fail to play a role in promoting health in school-aged children. Hence, such kinds of expert opinion were not considered. Some experts also suggested that the CAEPL should include the domain related to morals, owing to the national education policy. In line with national education goals and policy, it is recommended to include the domain related to morals. So far, however, there are well-accepted measures to assess morals in school-aged children, which impedes us from including the domain of morals. Moreover, an opinion indicated the removal of the domain of fitness and health. Indeed, in alignment with Whitehead, fitness and health is an essential element of PL [1,2]. Overall, despite some controversial opinions with constructive roles, the Delphi participants reached an agreement on the CAEPL; but most importantly, some of the valuable comments would pave the foundation for improvements in assessing PL in young people.

## 5. Strengths and Limitations

To our knowledge, this is the first study to develop comprehensive assessment and evaluation of physical literacy (PL) for Chinese children and adolescents, which extends knowledge in this field. Furthermore, this study delineated a picture that the developments of research regarding PL in China, and such experience may be beneficial to this field. As we invited international experts to participate in the development of the Chinese Assessment and Evaluation of Physical Literacy (CAEPL) and attained recognition, the CAEPL might be generalized internationally. However, some limitations in this study should be mentioned. First, due to some subjective reasons of the selected experts participating in the Delphi process, some experts did not respond in the subsequent rounds—this uncontrolled issue may negatively affect the CAEPL. Secondly, the final panel comprised experts from each CAEPL domain, but the number of the panel was not equally distributed, which may elicit bias for weights of domains of the CAEPL.

## 6. Conclusions

The Chinese Assessment and Evaluation of Physical Literacy (CAEPL) offers a model of physical literacy (PL) assessment that can support a measurement tool among Chinese children and adolescents. This study introduced the origin, development and implication of the CAEPL. The study contextualized the concept of PL by Whitehead in a Chinese context, which helps Chinese researchers to better understand the concept of PL, and links the concept with Chinese physical activity (PA)-related concepts. The CAEPL protocols are grouped within five primary domains: intention of physical activity (IPA); knowledge of physical activity (KPA); motor/sport skills (MSS); behavior of physical activity (BPA); and physical fitness (PF). The weights of domains of the CAEPL were also determined, with which MSS shares the largest weight, and then reflects the actual situation of Chinese school physical education (PE). Although the CAEPL provides an important and useful tool to measure and improve PL among Chinese children and adolescents, it is still a theoretical model. Thus, future studies concerning the feasibility, reliability, validity and sensitivity of the CAEPL should be conducted for its improvement in clinical settings, particularly in the context of school PE [37,38].

## Figures and Tables

**Table 1:** Characteristics of experts participating in each round of the Delphi process.

	Characteristics	Descriptions	*n* = 53		Characteristics	Descriptions	*n* = 34		Characteristics	Descriptions	*n* = 29		Characteristics	Descriptions	*n* = 23
Invited	Gender	Male	38	1st Round	Gender	Male	23	2nd Round	Gender	Male	20	3rd Round	Gender	Male	17
		Female	15			Female	11			Female	9			Female	6
	Country	Canada	5		Country	Canada	1		Country	Canada	0		Country	Canada	0
		China	33			China	27			China	25			China	22
		UK	5			UK	2			UK	2			UK	1
		USA	7			USA	3			USA	1			USA	0
		SWE	1			SWE	1			SWE	1			SWE	0
		NZE	1			NZE	0			NZE	0			NZE	0
		AUS	1			AUS	0			AUS	0			AUS	0
	Area of Expertise	PA	15		Area of Expertise	PA	8		Area of Expertise	PA	6		Area of Expertise	PA	6
		PE	45			PE	28			PE	25			PE	19
		PF	13			PF	9			PF	5			PF	5
		PL	30			PL	17			PL	10			PL	7
		APA	2			APA	1			APA	1			APA	1
		MD	5			MD	3			MD	1			MD	1

UK—The United Kingdom; USA—The United States of America; SWE—Switzerland; NZE—New Zealand; AUS—Australia.

**Table 2:** Domain of the CAEPL and its sub-domains and specific aspects.

Domain	Agreement%	Sub-Domains	Agreement%	Specific Aspects	Agreement%
IPA	89.70%	Intention of Physical Education Lesson	86.20%	Interest	89.70%
				Attitude	93.10%
				Motivation	86.20%
		Intention of participation in physical activity out of school time	100%	Interest	100%
				Attitude	96.60%
				Motivation	96.60%
		Intention of active play	100%	Interest	93.10%
				Attitude	93.10%
				Motivation	93.10%
KPA	100%	Kinesiology (basic)	82.80%	PA and muscle	82.80%
				PA and bone	86.20%
				PA and physical function	89.70%
				PA and cardiorespiratory fitness	93.10%
				self-rating for volume and intensity of physical activity	93.10%
				self-rating for Anthropometry	93.10%
				self-rating for fitness	96.70%
		Nutrition for PA and exercise	86.20%	Nutrients	86.20%
				Healthy dietary	93.10%
				Water, minerals and PA and exercise	86.20%
				PA Energy and PA and exercise	93.10%
		Health promotion and PA	93.10%	PA and health	100%
				Sedentary behavior and health	82.80%
				PA and body weight	93.10%
				PA and physical fitness	96.60%
				PA and motor skills	93.10%
		Safety/Injury/Damage of sport and exercise	93.10%	Principals of safety	89.70%
				Common injuries and prevention	96.60%
				Accidental damages and prevention	79.30%
				Cardiopulmonary resuscitation, CPR	93.10%
				Warm-up and cool down	96.60%
				Self-rating for level and capability for sport and exercise participation	96.60%
BPA	96.60%	PA and exercise	96.60%	Moderate to vigorous physical activity	93.10%
				Organized sports participation (physical education lesson during school time, physical activity except for PE in school, participation in sports clubs out of school)	100%
				Active play	100%
				**Active transportation**	68.90%
				**Screen-based time**	62.00%
				**Homework time**	55.20%
		Experience of sports games/events	89.70%	Participation in games/events within school (frequency and grades)	86.20%
				Participation in games/events between schools (frequency and grades)	75.90%
				Participation in games/event at regional or national level (frequency and grades)	75.90%
MSS	96.60%	Fundamental Motor Skill	96.60%	Locomotor	96.60%
				Object control	89.70%
				Body control	100%
		Specific Sport Skill	93.10%	football, basketball, volleyball, e.g., and other common sports-event for children and adolescents	/
PF *	79.30%	Physical function	/	BMI, Vital capacity (VC) of lung	/
		Strength		Pull-up, sit-ups	
		Power		50 m sprint, standing long jump	
		Cardiorespiratory fitness		Timed rope-skipping, 1000 m run, 800 m run, 50 m × 8 shuttles run	
		Flexibility		Sit and reach	

Agreement%—percentage of experts who selected ‘very agree’ or ‘agree’; if the agreement% is over 75%, it will be included in the theoretical model; IPA—intention of physical activity; KPA—knowledge of physical activity; BPA—behavior of physical activity; and MSS—motor/sport skills. Bold denotes that those specific items did not reach the agreement criterion, but this study kept them after inductive discussion. * denotes if PF was included in the theoretical model. All the measures to assess PF will be included as this study adopts a comprehensive and complete measure. /denotes no agreement%.

**Table 3:** Weights of domains and sub-domains of the CAEPL *.

Domains	Weight	Sub-Domains	Weight
IPA	0.1725	Intention of Physical Education Lesson	0.0448
		Intention of participation in physical activity out of school time	0.0490
		Intention of active play	0.0787
KPA	0.1623	Kinesiology (basic)	0.0393
		Nutrition for PA and exercise	0.0313
		Health promotion and PA	0.0427
		Safety/Injury/Damage of sport and exercise	0.049
BPA	0.2372	PA and exercise	0.1898
		Experience of sports games/events	0.0474
MSS	0.2701	Fundamental Motor Skill (for primary school-aged children)	0.2701
		Specific Sport Skill (for middle and high school-aged children)	0.2701
PF	0.1579	∆	/

* we reported the weights of five main domains and their subdomains of the CAEPL. ∆ denotes the weights of sub-domains are in line with the National Students Physical Fitness and Health Test (2014 Modified Version, NSPFHT). /denotes no weight.
